# Multilingual education: medical interns perceptions regarding the usefulness of non-mother tongue communications skills taught during the undergraduate curriculum

**DOI:** 10.1186/s12909-024-05414-1

**Published:** 2024-04-24

**Authors:** Ian van Rooyen, Joel Claassen, Natasha Moodaley, Gregory Doyle, Thuli Skade, Rae Nash, Sandile Gxilishe, Derek Adriaan Hellenberg

**Affiliations:** 1 Afrikaans and Netherlandic Studies, School of Languages and Literatures, University of Cape Town, Upper Campus, Cape Town, 7701 Western Cape South Africa; 2 Division of Family Medicine, School of Public Health and Family Medicine, Health Science Faculty, University of Cape Town, Observatory, Cape Town, Western Cape 7701 South Africa; 3 Department of Health Sciences Education, Health Science Faculty, Observatory, University of Cape Town, Cape Town, 7701 Western Cape South Africa; 4 African Languages, School of Languages and Literatures, University of Cape Town, Upper Campus, Cape Town, 7701 Western Cape South Africa; 5 Department of Clinical Skills, Health Science Faculty, University of Cape Town, Observatory, Cape Town, 7701 Western Cape South Africa

**Keywords:** Multilingualism in health sciences, Language learning, Multi-language use in the workplace, Additional language communication skills, Afrikaans, IsiXhosa

## Abstract

**Background:**

This paper investigates the perceptions of medical interns regarding the usefulness of non-mother tongue communication skills taught during the undergraduate curriculum at the University of Cape Town in South Africa. In 2003, the university decided to incorporate Afrikaans and IsiXhosa communication skills into the new MBChB curriculum in order to meet the Faculty of Health Sciences goals to promote quality and equity in healthcare, and to prepare graduating health practitioners for multilingual communities where they would be serving. Despite annual internal evaluations and reviews of the languages courses, the usefulness, if any, of the additional languages in the working clinical environment had not been determined.

**Methods:**

Data were collected during the second year of medical internship across a five-year period through survey questionnaires, as well as focus group interviews conducted in the Western Cape, South Africa. Surveys were conducted from 2009 to 2013.

**Results:**

The study shows that the usefulness of each of the probed categories was not consistent across both languages. The interns expressed a need for an overall improvement of the isiXhosa course offering, while the outcomes for the Afrikaans language were more positive across all categories except for cultural understanding.

**Conclusion:**

The study indicates a positive trend amongst the interns towards developing usefulness in communication skills in Afrikaans and isiXhosa to communicate with their patients.

## Background

The University of Cape Town (UCT) incorporated Afrikaans and IsiXhosa communication skills into the new MBChB curriculum in 2003 as part of the Becoming-a-Doctor (BaDr) course consequent to the Health Professions Council of South Africa (HPCSA) guidelines for the education and training of doctors in South Africa [1]. It was aimed at empowering medical students with work-based, career-orientated Afrikaans and isiXhosa, and had its first cohort of graduates in 2007. These graduates were serving their second year of internship in health complexes across South Africa at the time of the research.

UCT is situated in the Western Cape (WC) province and forms the backdrop of this study. It is recognized that, to practice medicine more effectively in South Africa, and the WC in particular, graduates need to possess some competency in the three official languages of Afrikaans, English and isiXhosa predominant in this province. Entry to UCT requires both written and oral competency in English. The South African National Census [2] of 2001 showed that 98.3% of WC residents are speakers either of Afrikaans, English or isiXhosa, while the census of 2011 [3] reported that 94.5% of WC residents are speakers either of Afrikaans, English or IsiXhosa. The WC Provincial Profile Community 2016 survey [4] found that collectively these languages are still spoken by 96,6% of households.

**Fig. 1:**
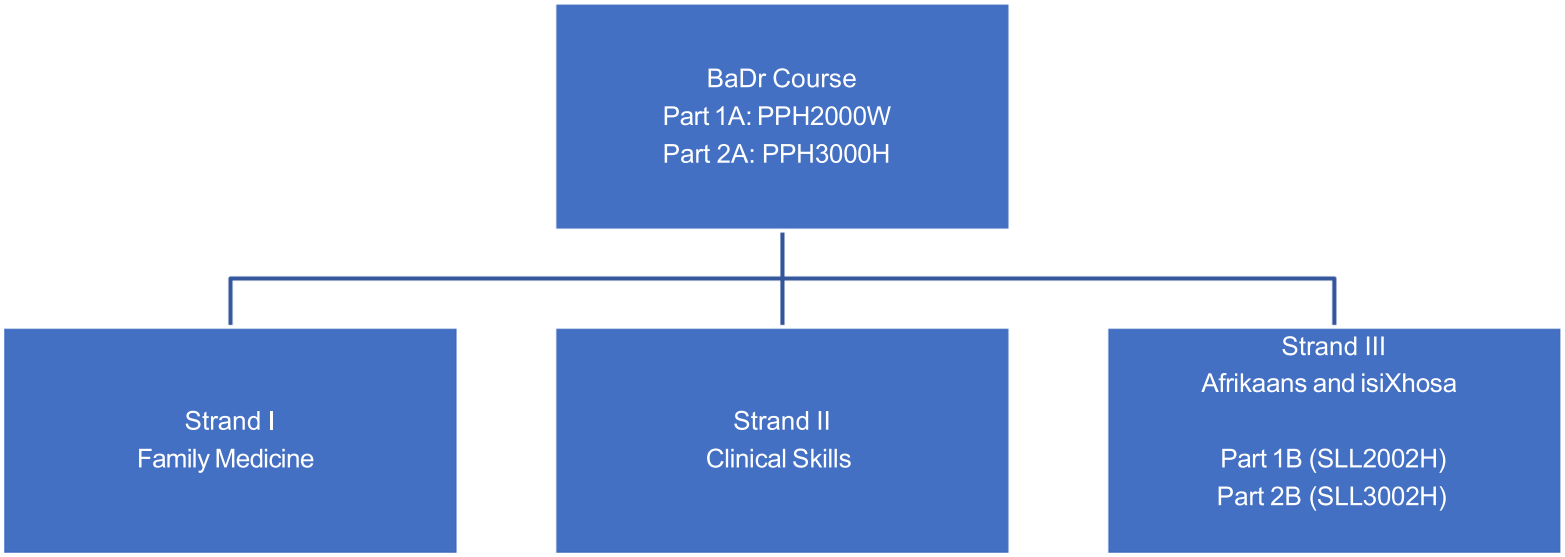
Diagrammatic structure of the BaDr course

The BaDr course runs over 18 months and is comprised of the constituent strands as per Fig. 1 below:

Each of the strands contributes equally (33.3%) to the course mark. Afrikaans and isiXhosa *(hereafter referred to as languages)* are weighted equally in strand III. The additional languages courses are aimed at providing graduates with at least a basic level of oral competency in communicating with patients who are mother-tongue speakers of these languages. Many improvements have been implemented over the years, including multimedia resources, as well as the Special Study Module (SSM), where participants live with Afrikaans or isiXhosa-speaking health practitioner hosts, who are affiliated with a Community Health Centre (CHC) for two and a half weeks of the four-week SSM period. Despite regular internal evaluations and reviews, this study is the first attempt in determining its usefulness in the working clinical environment. The study’s explicit aim is to investigate the perceptions of medical interns regarding the usefulness of non-mother tongue communication skills taught during the undergraduate curriculum at the University of Cape Town in South Africa.

In recent years, language has been identified globally as a barrier to providing effective patient care by the health care community. Deumert and Mabandla noted that in previous research [5], effective communication between the health professional and patient has long been recognized as being of diagnostic importance and therapeutic benefit. The manner in which language threatens effective communication between doctors and patients globally, and in South Africa in particular, has highlighted this barrier to healthcare. Schwei et al. contend that globally language barriers were reported to affect [6]:

i. quality of care in the health care systems around the world
ii. patients in their access to health services
iii. adherence
iv. quality of clinical care
v. and patient and provider satisfaction.

Since transitioning from an Apartheid state to a state of democracy in April 1994, the challenge to the South African health system is that there are 11 official languages. Historically, this largely monolingual nature of the health system must be understood in the context of the Apartheid system where there was a separation of races, and where black voices were historically suppressed [7]. This resulted in the majority of health professionals not speaking any of the indigenous languages [8].

Levin conducted a study in a tertiary paediatric hospital setting in Cape Town, which found that around 94% of interviews by health professionals were not conducted in the mother tongues of patients [9]. In addition, the interviews often occurred without interpreters. Van Rooyen, tracing the competencies of the 2011 SSM cohort, found that language immersion is a worthwhile investment in clinical competence as it fosters the independent acquisition of new knowledge, vocabulary, clinical competency, cultural awareness and proficient code-switching, where code-switching is the practice of alternating between two or more languages during a conversation [10]. In a study conducted in a rural clinic in the Eastern Cape Province, patients reported that together with cultural factors, language was a barrier to receiving effective health care [11]. Although once thought to be a solution, various studies have highlighted the limitations of using interpreters, which includes ethical issues around confidentiality. In addition, the concern over the competency of interpreters and patient satisfaction, have in recent years been questioned [12, 13].

Subsequent research has similarly suggested that both patients and health care professionals regard language as a barrier to the attainment of quality health care in South Africa [11, 14–19]. In particular, Deumert and Mabandla emphasised the language and cultural gap between doctors and patients, and stress the value of communication as an integral part of the practice of medicine [20]. Chima, evaluating informed consent in South Africa confirmed that “one of the major barriers towards obtaining valid consent …has been listed as ‘language difficulties’, ranked highest by 88% of doctors in this cohort…and the lack of interpreters by 82%… …it cannot be overemphasized that one of the major barriers to obtaining valid informed consent is the issue of language” [14]. Consequently, patients may not fully grasp the consequences of examination, or procedures to which they are consenting. Meuter et al. also stressed the impact of linguistic and cultural elements during language discordance and how it could affect health care provision [21]. Consideration should also be given to the impact of the unequal power relationships in a historically disparate South African landscape. It is, therefore, desirable, that the health professional is able to communicate in the patient’s mother tongue to deliver the best possible medical services.

Claassen et al. evaluated additional Afrikaans and isiXhosa career-specific, communication skills courses which were offered to doctors, nurses and general staff in two CHCs [19]. The study confirmed the excessive disparity in the language profiles of patient and healthcare providers in two impoverished communities in Cape Town.

### Interns and communication skills

In assessing different studies from around the world regarding medical internships, it becomes apparent that developing communication skills, and specifically skills in additional languages, is not necessarily a universal priority [22, 23]. Similarly, some significant South African studies regarding medical internships do not highlight communication skills or skills in additional languages either [16, 24–27]. In contrast, Sturman et al., found that junior doctors in Australia stressed the value of communication [28], while Tan-McGrory and Betancourt, in a study conducted in Israel, stressed that “ (i)f we are to truly deliver high quality, cost-effective care, meeting the needs of patients who experience language barriers during health care will be essential” [29]. The aforementioned studies therefore reflect that the need for additional language communication skills within an intern/junior doctor context is a global concern. Tavakol et al. in a study investigating interns’ knowledge of communication skills in Iran, pointed to the need “for integrating a communication skills course, which is linked to different ethnic and religious backgrounds of interns” [30]. It is thus acknowledged that communication skills courses must account for the cultures of different patients, while language is widely regarded as one of the most important cultural markers.

In South Africa, Classen et al., stated that “…intern training provides opportunities to further develop skills in communication and teamwork as they were equally important in the delivery of quality services” [31]. Even so, Mofolo and Botes, conducted a study that evaluated factors influencing the experiences of final-year interns in the Free State Province where respondents reported that “(t)he negative aspects [of their experiences] included…language issues” [32].

## Methods

This was an exploratory mixed-methods study comprised of a questionnaire survey and semi-structured focus group interviews. The design was open and flexible, and was regarded as appropriate, as the topic is poorly understood, with limited scientific data. The survey targeted MBChB students who graduated from 2007 to 2011 to evaluate their perceptions regarding the usefulness of non-mother tongue communication skills taught during the undergraduate curriculum. Sixty five graduates were surveyed during their second year of internship from 2009–2013.

### Survey questionnaire

A draft survey questionnaire was developed by the researchers as the most appropriate method of communicating with the interns. The background and aim of the project were explained to the interns and they were informed of their right not to participate if they so wished. The questionnaire was piloted on a small group of interns during one of their regular seminars. The draft questionnaire was commented on by the group and their feedback led to some changes before it was finalised and converted to an electronic version.

Contact details of graduates were obtained for each corresponding cohort by the undergraduate office in the Faculty of Health Sciences at UCT. Updated details were also obtained from the Tygerberg Hospital management which is one of the tertiary teaching hospitals at which UCT graduates would do their internship. The contact details were entered into an Excel spreadsheet. Electronic questionnaires were sent first via e-mail and those interns who had not responded after two weeks were sent hard copies of the questionnaire by post. SMS reminders to complete the questionnaire were sent simultaneously. The excessive workload of interns in South Africa has been negatively highlighted in the media, where 60 h per week workloads have drawn much scrutiny. Against this backdrop, the response rate to this study was 5% relative to the 1400 survey requests sent out.

Completed questionnaires were received at a central office at UCT. Electronic response data were captured by UCT’s Learning Management System, VULA, and exported as an Excel spreadsheet. The responses from hard copies were captured manually into the same Excel sheet. The data were analysed, cross-tabulated, synthesized and patterns were identified. Tables and graphs were produced in Excel.

### Focus groups

Focus group participants were recruited from the Groote Schuur and Tygerberg Hospital tertiary teaching complexes. The recruitment process ensured that interviewees were available and that their participation was consentual and voluntary.Interviews were conducted by experienced interviewers and information was obtained using a standardized semi-structured format. The participants had, within the framework of the opening questions, the freedom to explore their own ideas and suggest new topics which may have been of importance to the opinions expressed. The opening questions were:

- ➣ What are your thoughts on the usefulness of the language learning you had in the BaDr course as applied in your clinical work as an intern?
- ➣ If you were the designer of the course, would you structure the course differently with regard to content and method of instruction?
- ➣ Any other comments with regard to the course, facilitators or venues used?

The interviewers summarised, reflected, stimulated and asked for clarification where necessary. Focus group discussions were planned with a sample of interns from the WC during 2009 and this process was intended to be extended to the other provinces between November 2009 and April 2013. Due to funding constraints, we were unable to conduct focus groups outside of the WC. WC interns were sampled and interviewed as from 2010.

The intention was to have at least 8 participants in a group, which was not always achieved. Due to a low participant uptake, the study relied on convenience sampling. Five focus groups were conducted, each group consisted of a varying number of participants (7; 5; 5; 4 and 2) who were randomly chosen from a list of UCT graduates working at the Groote Schuur and Tygerberg Hospital tertiary teaching complexes. Participation was on a purely voluntary basis. Participants were offered an opportunity to read the transcribed data for informant validation purposes where focus groups had been conducted and to feed back to the researchers where any inaccuracies had been identified. All interviews were conducted in English and no interpreters were required. There was no observer or video camera present. However, the interviews were audio-recorded and transcribed in English with the verbal consent of the participants. Verbal consent was also sought from the participants to publish the findings in journals and to present them at conferences.

Themes and sub-themes followed content analysis and were systematically coded and indexed into these categories using NVivo software. Interpretation was constrained by intimate data knowledge, categorization, flexibility and counter-analysis, as well as the exploratory nature of the study. There was no hypothesis and data were presented as found.

## Results & discussion

The findings of this study give us insight into what interns said and could do with regard to languages communication in the workplace. It is noticeable from the probed categories below that what interns reported about Afrikaans, was not necessarily congruent with what was reported about isiXhosa. The fact that more interns professed some proficiency in Afrikaans relative to isiXhosa upon entry into the BaDr course, seems to be consistent with the general trend of respondents reporting higher levels of proficiency in Afrikaans in identified tasks in the clinical setting than in isiXhosa. In addition, isiXhosa and Afrikaans are non-cognate to each other, in that they are linguistically unrelated. IsiXhosa is an Nguni Bantu language, while Afrikaans is an Indo-European language, which has different linguistic forms in respect of syntactic, morphological and semantic relations. Consequently, it is challenging for Indo-European speakers to acquire isiXhosa as adults, and vice versa. In addition, Afrikaans is widely taught in schools and a significant number of the participants would have done Afrikaans at school. Relatively less students would have done isiXhosa at school.

In order to successfully investigate the usefulness of acquired non-mother tongue communication skills in the workplace, insight is required into the contextual factors that influence the interpretation of the data.

A crucial aspect is an awareness of the linguistic profile of individual participants. Most students registered for BaDr are bilingual. Various mother tongues are represented by the target group and hence a range of permutations are observed with respect to the first and second languages which are spoken. The language combinations interns used most often were English/Afrikaans (42%), English/isiXhosa (6%), Afrikaans/English (3%) and isiXhosa/English (2%). English is the most used language in any of the combinations.

Across the years of the study, an average of 15% of interns rated their speaking ability as beginners in Afrikaans, while 61% of them stated they were beginners isiXhosa. Interns who identified themselves as beginners in either Afrikaans or isiXhosa are rated equivalent to the Common European Framework pre-level A1, American Council on the Teaching of Foreign Languages (ACTFL) Novice Level or Defence Language Proficiency Test (DLPT) level 0 or Interagency Language Roundtable scale (ILR) Level 0. On average, 8% of the interns reported that they were complete beginners in both languages. This detail may have influenced the degree to which interns embraced opportunities to develop oral competency in the target language, initially during the undergraduate years, and later during their period of internship.

The linguistic profile of patients at the clinical sites where interns practised may consequently also have influenced responses. The reported mother tongues of all the interns who completed the survey, in descending order are: English (71%), Other (23%), isiXhosa (3%) and Afrikaans (3%). The order, however, of the languages as reported spoken most often are; English (86%), Afrikaans (5%) and isiXhosa (3%). In contrast, the languages commonly spoken by patients were Other (37%), English (25%) followed by Afrikaans (18%) and isiXhosa (17%).

The statistics above indicate that there is a misalignment between the languages which are spoken most by doctors and those by patients. Since patients are more likely to speak a language other than Afrikaans, English and isiXhosa during their encounter with a doctor, it can be deduced from the data that conversationally doctors are migrating to English as the Lingua Franca to facilitate meaning during the consultation.

The following section highlights what interns’ responses were to select questions from the study questionnaire about their communicative abilities in the respective languages. When reference is made to the positive spectrum of responses, the ‘agree’ and ‘strongly agree’ categories are viewed cumulatively. Similarly, the negative spectrum relates to the cumulative responses from the ‘disagree’ and ‘strongly disagree’ categories.

### Interns can understand the cultural context related to Afrikaans and isiXhosa better

**Fig. 2:**
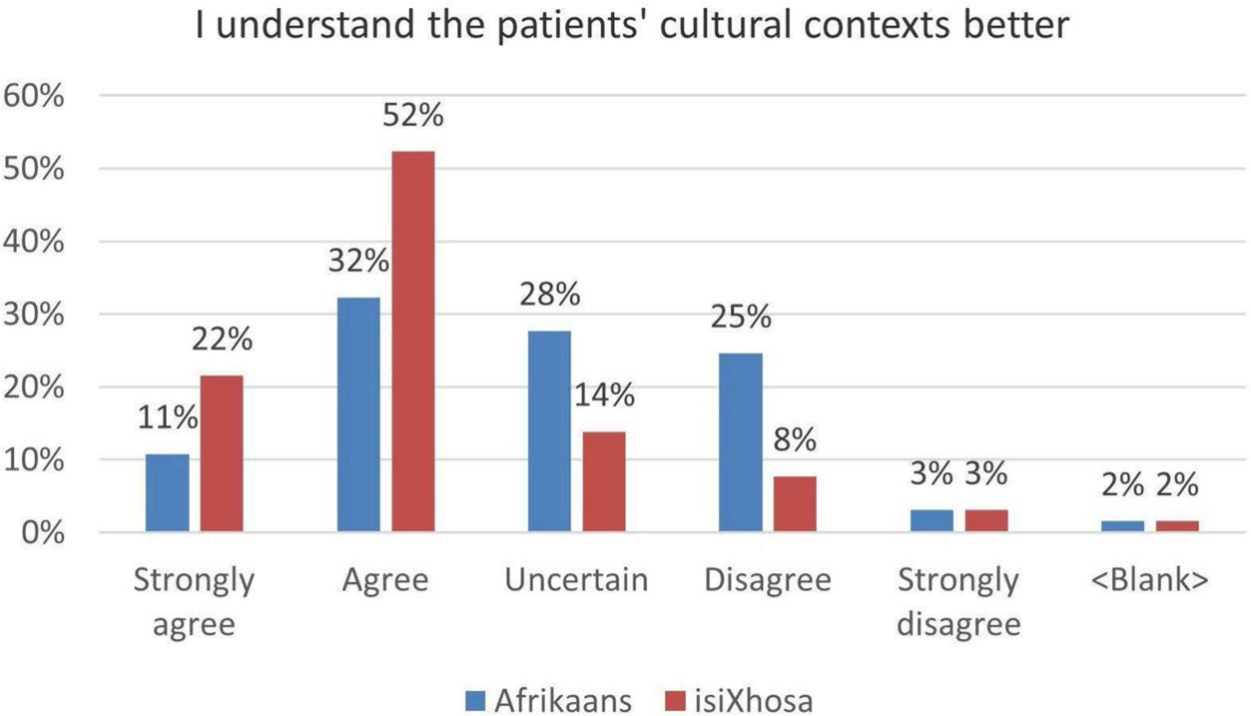
Reported understanding of Afrikaans and isiXhosa culture

A response of 43% in the positive spectrum in this category confirms the complexity of understanding Afrikaans culture (Fig. 2). Unlike many other languages around the world, where a language is often intrinsically linked to a homogeneous culture, the same cannot be said for Afrikaans, where it is difficult to define a typical Afrikaans culture. Even as diverse linguistic backgrounds of groups contributed to developing 17th Century Dutch into Afrikaans, contemporary Afrikaans still has speakers from diverse cultural backgrounds. In the BaDr course, students are taught Standard Afrikaans, while an awareness is also created of some other common varieties of Afrikaans such as Cape (“*Kaapse”)* Afrikaans. Confidence in cultural awareness is therefore only acquired through frequent interactions with the targeted linguistic and cultural demographic, as the study participants were doing as interns within CHCs in the WC.

The notion and understanding of isiXhosa culture is integral to the communication course which is subsumed in the MBChB degree. The study participants interacted with mother tongue isiXhosa-speaking patients which presented them with the opportunity to engage with the cultural aspects that they learnt during the undergraduate years. Amongst other cultural elements, the interns needed to apply their knowledge of taboo words, display respectful body language and eye contact, and demonstrate an understanding of the role of traditional beliefs and religion. An average response of 74% (Fig. 2) in the positive spectrum in this category demonstrates an appreciation of this vital issue.

### The interns can greet patients fluently and understand their replies

On average, 91% of interns reported that they are able to fluently greet their patients in Afrikaans, and 88% are able to understand patient’s replies to greetings. Eighty-nine (89%) percent can greet the patient fluently in isiXhosa while only 68% of interns were able to understand the patients’ responses to their greeting.

Meeting and greeting effectively is one of the competencies that students have the most opportunity to practice during the MBChB degree. It is a criterion which is tested at all BaDr assessments and is an ability practised during all clinical visits throughout the students' course of study. The action of meeting and greeting includes: self-identification as a doctor, addressing the patient appropriately and respectfully, establishing whether the patient is comfortable, enquiring after the patient’s well-being and providing a statement of confidentiality. Interns were more confident in understanding replies to greetings in Afrikaans than the act of greeting itself. What this suggests is a development in comprehension skills. There is no way of predicting beforehand how detailed a patient’s response would be after enquiring into their well-being. The more elaborate the response, the greater the demand on the intern’s comprehension ability. What the survey therefore reveals is that interns are comfortable in initiating a therapeutic relationship in Afrikaans and demonstrate a great level of comfort doing so in the patient’s mother tongue.

Furthermore, the forms of greeting are one of the first, and most important elements, taught during the Languages courses, particularly as it ties in on cultural levels. On the one hand, greetings establish the bond between the doctor and patient. On the other hand, it lays the foundation for the quality of the relationship, how interns participate in greeting the patient, and how their cultural knowledge is reflected through their verbal utterances. Not greeting an isiXhosa patient with the appropriate acknowledgement of their social standing, leads to alienation and displays disrespect to the patient. An average response rate of 89% within the positive spectrum indicates that participants are comfortable in greeting in isiXhosa, and that they recognise the vital role of greeting the isiXhosa patient correctly. The importance of Interns introducing themselves fully by name and occupation and clarifying their role, contributes to putting the patient at ease, a competency that the medical interns have grasped.

After at least two years of isiXhosa communication skills tuition, one would expect interns to display a higher understanding of responses to greeting. However, from a cultural point of view, when first greeted by medical practitioners and asked about their welfare, isiXhosa-speaking patients may immediately start speaking about their ailments, their symptoms and its influence on their daily functionality. Although patient-centred health care practice is common in South Africa, a three-stage assessment methodology underpins the medical practitioners’ assessment of a patient’s primary presenting complaint and determining its diagnosis. This means of patient assessment [Clinical, Individual and Contextual assessments], which aims at utilising a subtle structure, could at times elicit isiXhosa responses like [“*Ndiphilile ngaphandle kwenkosikazi egulayo ekhaya”* meaning ‘*I’m fine* except (for) my sick wife at home.’] or [“*Inkomo zam zifile yimbalela” meaning ‘My cattle died from (the) drought.’*]. This may make it extremely difficult for the burgeoning isiXhosa communication skills of interns to understand the scope of the responses. If the responses contain elements that straddle the clinical, individual and contextual categories, interns may feel overwhelmed, as patients may be discussing symptoms and their impact, in a non-linear manner, or with language that the interns do not know yet or feel comfortable with. This may contribute to interns hearing responses that they did not expect, or be unrelated to the primary presenting complaint, which may reflect a lack of medical competency. Together with this possibility, the interns however must respect the cultural elements of sharing, which may make it difficult for them to identify the patient’s needs, thereby affecting their own level of understanding.

### Interns can ask relevant questions to establish the presenting complaint

In this category, 80% could ask the relevant questions in Afrikaans to establish the primary presenting complaint. The proportion of respondents who reported either an inability or uncertainty to establish the presenting complaint in Afrikaans could be linked to patient responses which include conditions that are not specifically covered in the BaDr curriculum. Interns may still grapple with the skill or struggle to retain vocabulary that they had learned during their undergraduate years. Without an understanding of what a patient’s presenting complaints are, it is more challenging to explore these.

Relative to Afrikaans, a larger proportion of interns indicated an inability (21%) or uncertainty (12%) to establish the presenting complaint in isiXhosa. These results should be interpreted with the fact that respondents last received formal isiXhosa communication tuition at least four years prior to the study. There may be a measure of attrition when considering their retention of vocabulary, if there was no continuous application of communication skills in the interceding years. The measure to which interns are familiar with the possible responses to questions that are posed to patients, may affect the responses in this category. Another contributing factor may be the amount of time interns invest in self-directed language learning.

### Interns can ask relevant questions to obtain a history from a patient and understand patient responses during history taking

Since history taking is central to every case in medicine, consequently it becomes second nature to conduct it in any language. From the interns' responses, 73% agree that they can ask relevant questions to obtain a history from a patient in Afrikaans. Furthermore, 72% agree that they understand patient responses in Afrikaans while a history is being taken. Regarding isiXhosa, 46% of interns agreed that they can ask relevant questions in history taking. However, 69% felt that they are either uncertain or unable to understand patient responses during history taking.

The Afrikaans statistics for this category suggest that active language learning is manifested during the history taking component of the consultation, which is fundamental to the process of making an accurate diagnosis. While history taking demands active speaking and interrogation skills, the understanding of patient responses requires a different set of linguistic skills, namely, listening and comprehension. The results for Afrikaans therefore suggest that respondents are as confident listeners as they are speakers.

The isiXhosa responses in this category may be attributed to the fact that new vocabulary is required for history taking. It therefore becomes imperative for students to learn the vocabulary across every formal lesson. Several language learning factors may contribute to this lack of confidence, namely, the time needed to learn vocabulary, the regularity of language classes and time spent outside of class in language learning. The structuring of sentences could also deter interns from asking enough history taking questions, to extract the appropriate information about the symptoms from the patients in isiXhosa.

Thorough comprehension of the numerous answers to history-taking questions requires mastering isiXhosa tongue-clicking. In this context, a substantial vocabulary is required. Furthermore, it is often the case that isiXhosa-speaking patients do not restrict their responses in a way that beginners in the language can comfortably comprehend.

### Interns can give the patient necessary instructions to conduct a clinical examination and understand the patient’s responses during the examination

**Fig. 3:**
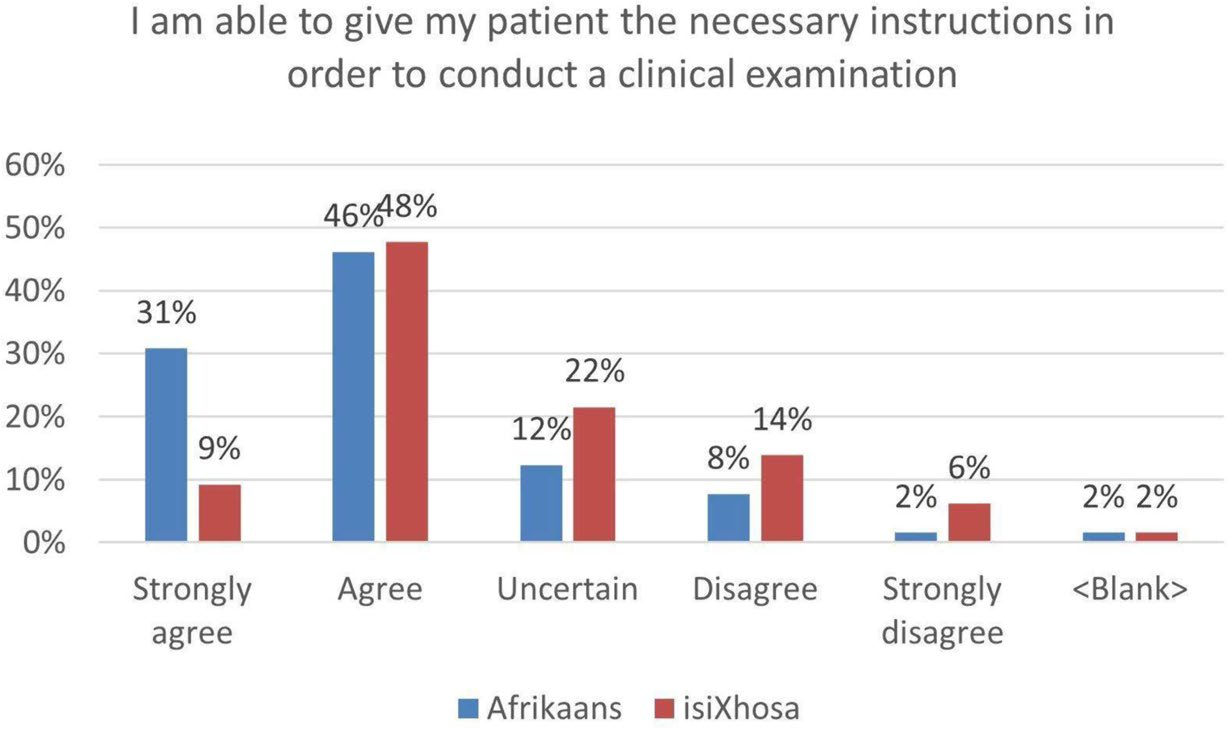
Reported ability to give instructions during a clinical examination

The following graph shows that 77% of respondents agreed that they could give the necessary instructions in Afrikaans to conduct a clinical examination (Fig. 3). A total of 80% believed that when patients responded to these instructions, they were confident that they understood the reply. Similarly, 57% agree that they are able to give patients the necessary instructions in isiXhosa.

In terms of conducting a clinical examination in isiXhosa, interns appear to have been more comfortable in giving necessary instructions in the early years of study. The interns are guided by the required clinical procedure, which informs the instructing process. In the formative years of the isiXhosa course, the focus fell on the skill of asking questions and giving instructions. Due to limited vocabulary, the development of comprehension skills were delayed. As time progressed, students were required to increase their repertoire of isiXhosa speech acts (giving instructions relating to numerous procedures; having an extended conversation, giving advice, and referrals). The increase in workload and grammatical complexity may have led to a reduction of confidence and minimised the retention of vocabulary.

**Fig. 4:**
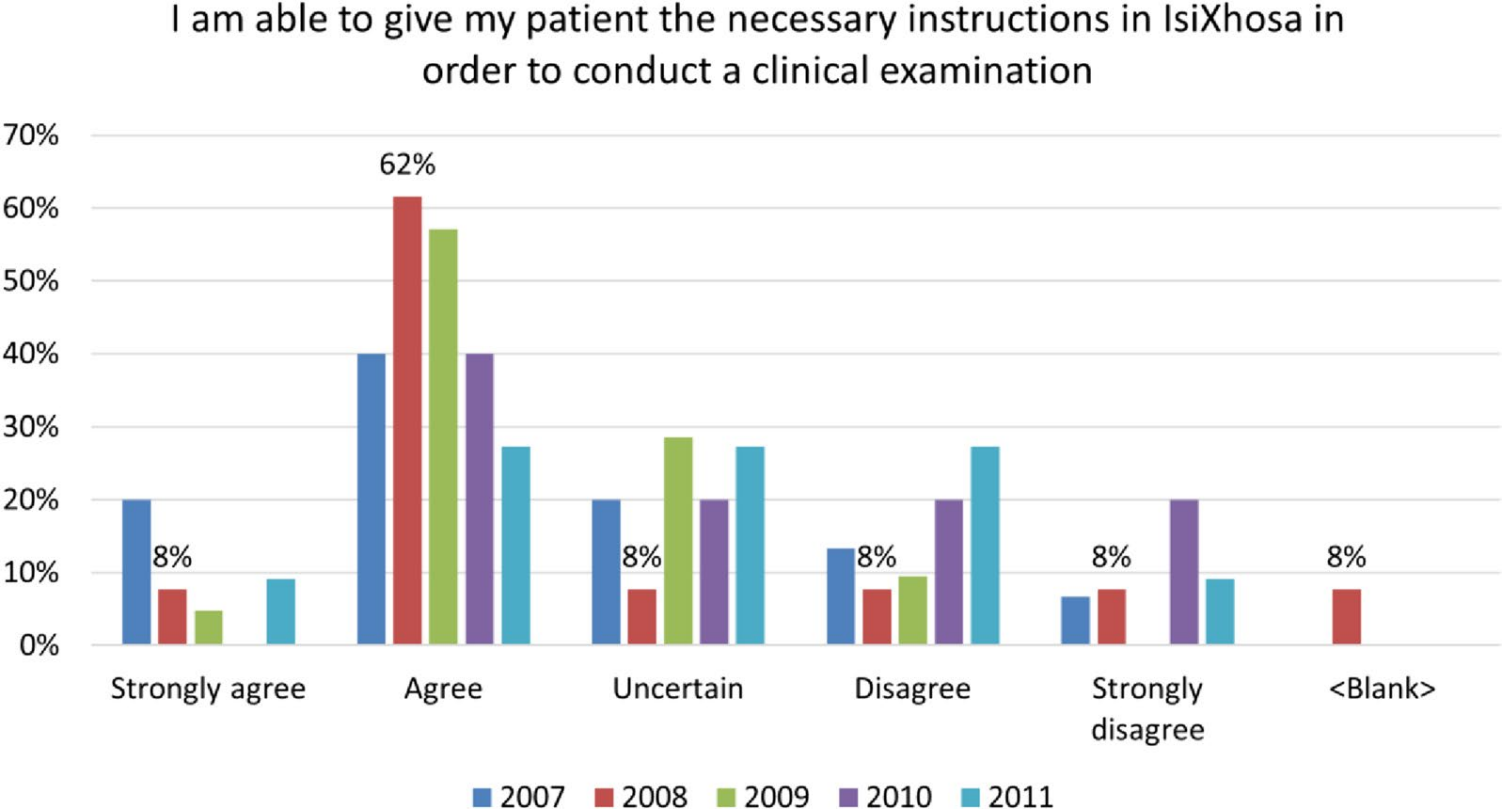
Reported ability to give instructions in isiXhosa during a clinical examination per cohort

The intern feedback insofar as they expressed their ability to understand patient responses in isiXhosa (34%) during the clinical examination, confirms this. Figure 4 shows higher levels of comfort in the years where comprehending responses was not part of the course (2009 and earlier). In 2010 and 2011, where this skill became part of the course, the percentages dropped. The coursework was then amended to include more dialogues, which served to stimulate comprehension.

### Interns can explain to their patients about their illness and assess their understanding thereof

**Fig. 5:**
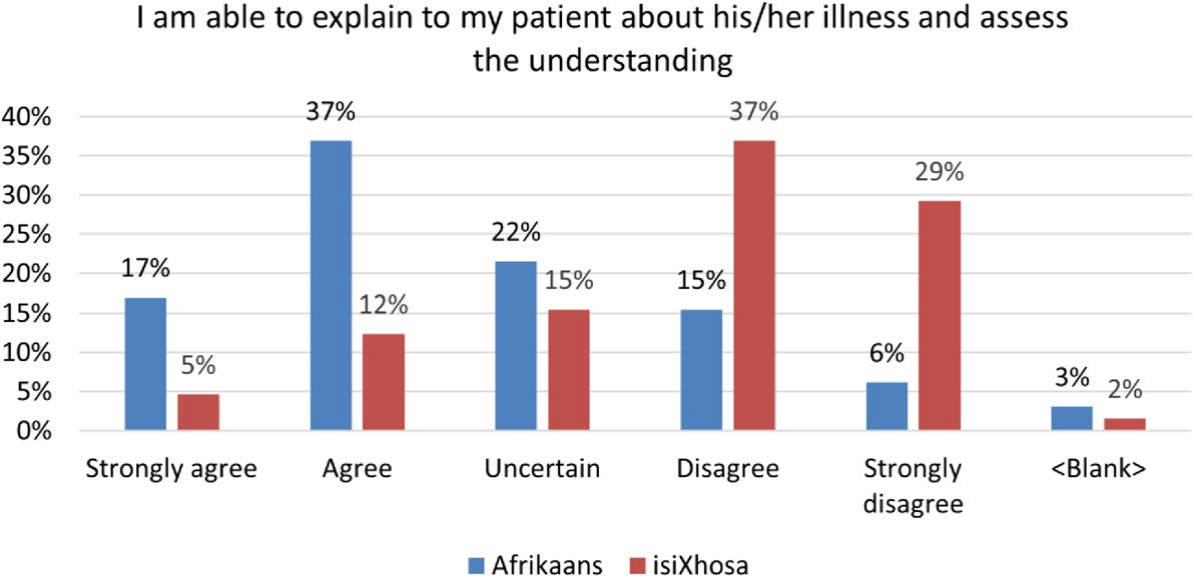
Reported ability to explain and assess understanding of patients’ illness

According to the graph below, only 54% of interns agree that they were confident in their ability to counsel patients about their illness in Afrikaans and assess their understanding thereof (Fig. 5). For isiXhosa, 66% disagreed with the same statement.

Sharing information on a specific illness in any language challenges the speaker’s communicative ability, as it requires the synthesis of a medical register into one which befits a more colloquial style. For this reason, it is expected that communicators with a lower level of oral proficiency will find this stage of the consultation more challenging. The MBChB curriculum equips graduates with the knowledge to counsel patients on a myriad of illnesses. The BaDr course offers multilingual exposure to systems covered in the Year 2 and 3 curricula; the general examination, the cardio-vascular, respiratory and neurological systems, as well as HIV/AIDS, anaemia and jaundice. Therefore, where illness is concerned, the multilingual clinical ability in many interns is limited.

Making a diagnosis and the associated vocabulary and new knowledge such as *breaking bad news* was a vital addition to the isiXhosa courses after 2007. Subsequently, the negative response is to be understood, as respondents do not feel that they possess the vocabulary to understand the range of possible isiXhosa responses.

### Interns can explain to the patient how to use their medication and assess their understanding thereof

**Fig. 6:**
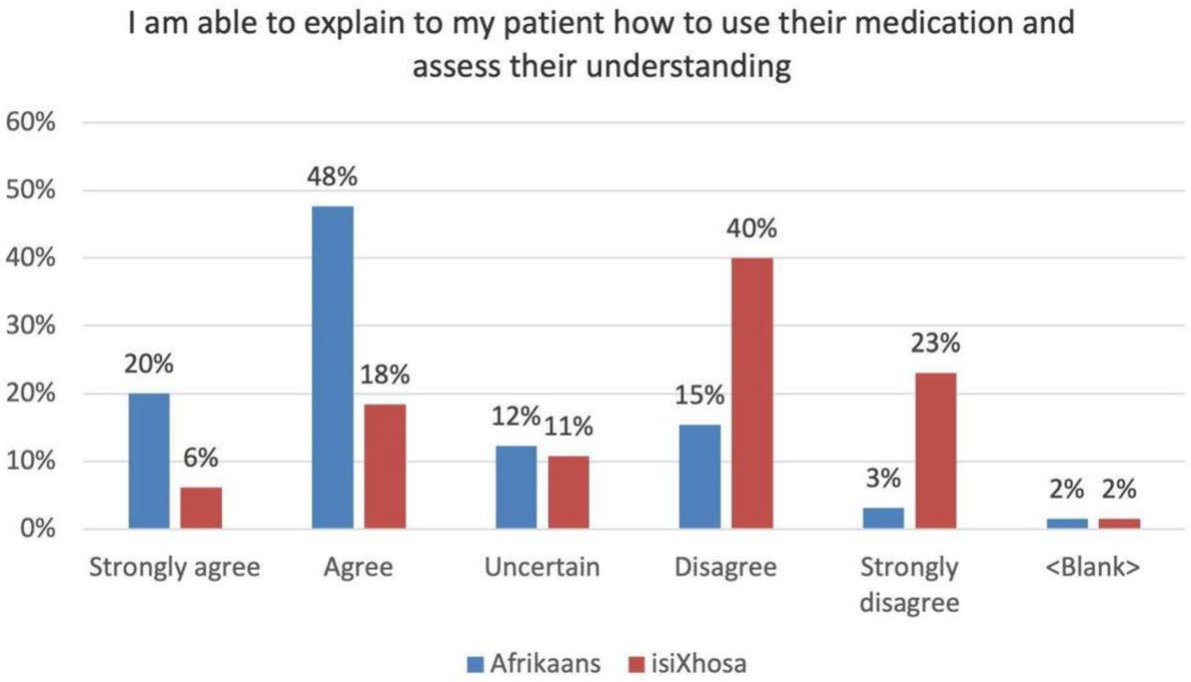
Reported ability to explain how to use medication

Despite the uncertainty of some respondents, 68% agreed that they had the capacity to explain to a patient in Afrikaans how to use prescribed medication, and to assess the patient's ensuing understanding (Fig. 6).

Prominent themes covered in the *Languages* strand, include establishing the presenting complaint, taking a relevant history, discussing the nature of the illness and its management. Regarding the prescription of medication, students are taught only a generic vocabulary. They do not possess the terminology to discuss types of medication, how frequently it should be used and what its indications are. Here too there is a possible terminological deficiency as only illnesses covered in the Family Medicine courses were taught in the Languages courses. The graph highlights a comparison of the context relevant to this category with the preceding one (Fig. 5). It reveals an emerging trend in the Afrikaans component of this survey: a gap in multilinguistic content knowledge is directly proportional to an increase in an ‘uncertain’ response in the associated survey question. Multilinguistic content knowledge is having the necessary vocabulary to communicate on information relevant to a specific domain. In this case there was a gradual increase in the number of respondents who were ‘uncertain’ (12%) about their ability to talk about the prescription of medication. In the previously reported category of understanding the Afrikaans cultural context better, the uncertain response was 28% (see Fig. 2), which reinforces the assertion that it is linked to a gap in multilinguistic content knowledge.

Of the interns, 24% reported that they would be able to explain the use of medication in isiXhosa, which invariably involves the use of different types of clicking in isiXhosa (Fig. 6), which students anecdotally report as having a measure of difficulty. If the interns are unable to produce isiXhosa clicks appropriately, the pronunciation would alter the meaning of the words. Thus, misunderstood instruction may then have an adverse effect on the communicative process, as well as potentially harm the patient’s health. It is thus understandable that collectively 63% of respondents report that they would be unable to explain medication use.

### Interns can counsel the patient on basic health promotion

**Fig. 7:**
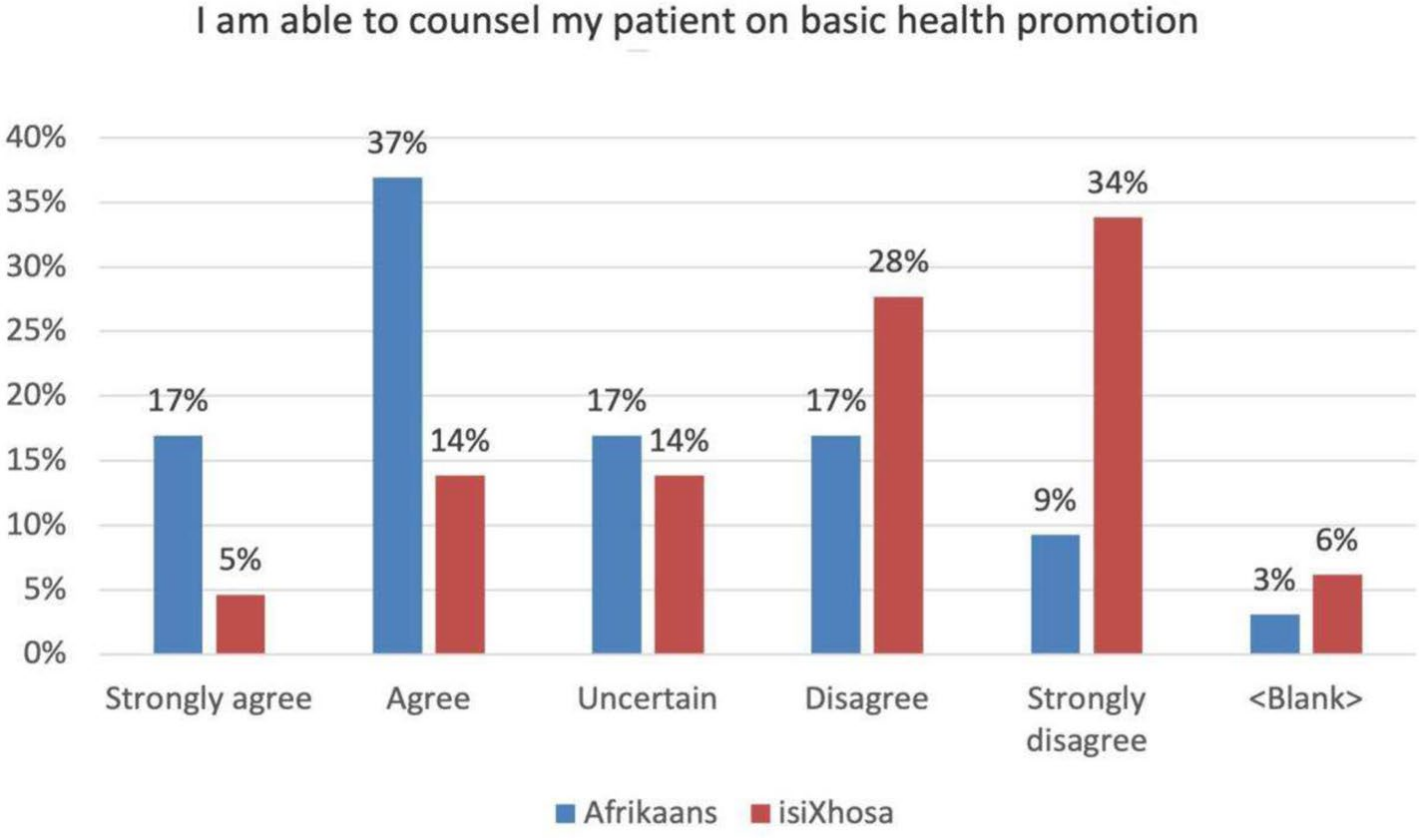
Reported ability on how to counsel patients on health promotion

Health promotion is not an explicit outcome of the communication skills courses. Consistent with the previously predicted trend in Afrikaans, Fig. 7 shows an increase in the ‘uncertain’ response, where 17% of interns identified themselves as such. Of the respondents 54% agree that they were able to counsel their patients on health promotion, while effectively 29% felt that they were unable to. It must be remembered that a large percentage of respondents received some tuition in Afrikaans at school prior to commencing the communication skills courses which meant that their starting point for the development of Afrikaans was different to that of isiXhosa.

In isiXhosa, a low score in this category may be expected in participants who are learning a language non-cognate to their mother tongues. According to the data only 19% of respondents agreed that they could offer appropriate health promotion in isiXhosa. Health promotion communication places immense linguistic demands on a speaker who has limited proficiency in the language. It requires not only encyclopaedic knowledge of the topic, but also the ability to synthesise and correctly apply the appropriate grammatical, phonological, morphological and lexical knowledge. The statistics in this category therefore suggests that this is one area in the BaDr communications course that requires attention. The challenge however does not lie in expanding the existing course, but rather identifying where and how additional information is included in a demanding curriculum delivered under tremendous time constraints.

### Qualitative focus group findings

Participants in the focus group interviews agree that even learning the basics in Afrikaans and isiXhosa languages during their medical training has helped them with their present clinical work as evidenced by the following comments:

*…I needed to like use what basic knowledge I had. I find that in that way the introduction was useful.*

*You’re not going to know all the Xhosa that you learn at med school, internship you forget like 90% of it, but most of it like isifuba (chest problem), comes in handy.*

*I think it has made us better doctors. And I think that it was, the language that ...at first are very limited and usually only applicable to medicine so I can’t ask a lot of other things other than direct medical questions, but it is enough that I can get through if I am stuck without a translator.*

Although some interns do not feel that they mastered a high-level proficiency in the languages, they do believe that the communications skills in the additional languages assisted them with the ability to do their jobs more effectively as interns. As one participant states above, *I think it made us better doctors*. In addition to the curriculum assisting with vocabulary and extensive knowledge of question formation that helped with history taking, it also created an atmosphere where patients were more at ease seeing their doctor.

*Yeah, I think it was overall very helpful. The look on people’s faces when they’ve been frustratedly trying to communicate with people, and you just say one sentence, and they all laugh, all of them will laugh...Aaah! And you just suddenly become the most amazing person in the room. It’s a lovely feeling just to think that they know you actually made the effort.*

*I think it is actually quite amazing when you walk to a patient and they see you and you greet them, and they answer you. And then they immediately almost want to speak to you and that the fact that you are not that articulate doesn’t actually matter.*

From the group discussions there appears to be a general sense that the introduction of languages into the medical curriculum has been helpful in assisting with the interns' clinical work, as they are able to assist patients. It also fosters better relationships with patients when they access the public health care system, as evidenced from the participant comments above.

### Curriculum design feedback

As Tyam and Mohamed et al. contend, there was also critical feedback from the respondents with regard to the course design, which had been updated after the annual course reviews [33, 34]. Although Beginners receive independent classes, respondents felt that the basic grammar of the languages was not optimally taught, specifically within the isiXhosa teaching. Many respondents felt that they had their steepest learning curve with isiXhosa. For most it was not a subject at school, unlike Afrikaans, and therefore they needed to be grounded in the basic grammar, which would require extra tutelage.

The universal feeling is that the way the isiXhosa course was structured could be improved to incorporate greater learning support with basic grammar, syntax and noun classes as important starting blocks that were not included when the course was introduced to them.

*I suppose, once you get the vocab down… then you will have to start putting sentences together. But like keeping it basic. And just making sure the sentence is built up gradually word by word added together. And at the end you say [a] sentence and you actually know what you are saying.*

*No, I don’t think roleplaying and that will actually help, it’s more the teaching us how to construct a sentence basically or understanding it. I think it would take more than probably the little time that we have in order for us to learn a language.*

Students want structured, scaffolded language instruction rather than learning phrases where they were unable to use the building blocks of the language to explore and experiment with the languages themselves.

*I was also thinking more in terms of the personal responsibility. If you learnt the basics, more basic like language structure and stuff then you have more something to work on in terms of building up your own skills as opposed to just learning sentences.*

This participant compared her learning of the language with what she remembered from school and how her school experience was more useful.

*But I remember thinking at the time that when we were learning at school, we learnt it more in terms of principles, whereas at varsity I found it was a lot of trying to memorise long sentences, you did not even know what the different parts of the words meant. ... And I remember thinking that I much preferred it the way we did it at school.*

While interns, who were Beginners, were able to communicate some ideas and commands to patients in their mother tongue, one respondent said things usually “fall apart” when the patient would respond, which points to a greater need for vocabulary and semantics particularly for the Beginners. Some interns report calling on other medical staff, such as nursing staff, students, other patients, and often porters to assist with translations, which however provides adverse challenges and ethical concerns.

### The value of early exposure and application during the undergraduate years

At no time did participants express that they had reached a point where the knowledge of Afrikaans and isiXhosa had been saturated, and the only way they might improve their proficiency levels was through practice.

*…I mean we are there for six years. So, in that time, if the training was started at the beginning and ended at the end and became more medical focused only at the end and beginning started very generally with conversational languages, then you would have had six years of training and you would have developed the breadth of the language that is necessary to understand...*

*…I think I would stress the clinical part and incorporating the language in the clinical part because that’s the only way to remember.*

Participants also highlighted the importance of getting early exposure to the purpose of learning the language. They agreed that they were not motivated to learn the language as students, as their focus was medicine. The interns admit that they had not realised that languages would assist them in their careers, allowing them to be *better doctors*. This participant suggests that early exposure to a clinical setting might emphasise the importance of learning languages while studying medicine:

*Perhaps even taking just like one trip with the people right at the beginning to Gugulethu and put in small groups with a consult doctor at a time. And then just letting the consult doctor continue doing the normal thing, and at the end maybe the doctor can just explain to them because we all feel like that because we are working there now. You know, just so they can see the frustration of a patient being on anti-hypertensives...*

It appears the interns felt that if they are able to understand the importance of language coupled with clear, realistic sets of outcomes for their learning, there may be better participation from them during their undergraduate years. It was suggested that the only way they might improve their proficiency levels was through integrated practice during the clinical years of undergraduate study.

However, in a study of the undergraduate student perceptions of the communication skills courses at UCT [34], only 52% indicated that they worked on their isiXhosa (in varying amounts of time), while 53% indicated that they worked on Afrikaans (in varying amounts of time) on a weekly basis. This ties into the focus group comments, that as undergraduate students they needed to invest more time on the additional languages and explains why many interns felt a lack of confidence in using the additional languages to communicate.

## Limitations of study

The limitation of the study has typical features of a longitudinal study in that the response rate was low, as follow-up with participants after leaving the university was nearly impossible to maintain. It was thus difficult to guarantee participant respondents who were predominantly in the WC, the primary space in which graduates were expected to practise after graduation. Some respondents may not have been within CHCs where they were able to utilise the additional language communication skills. The study could therefore not determine what the nature of their clinical exposure would have been had they not been placed in the WC. An annual review of cohorts, therefore, should be a future consideration.

We were able to establish the degree of individual reported competencies, but we could not determine the full impact thereof due to the placement of interns in provinces where the need for interaction in the target language is minimised. In order to establish full impact, a follow-up study of UCT medical graduates practising in the WC would be the logical next step.

## Recommendations

When this study was initiated, UCT was one of the first institutions in South Africa with a comprehensive additional language programme embedded into Health Sciences curricula. The University of the Western Cape, Stellenbosch University, The University of Kwazulu Natal and The University of Pretoria amongst others have also launched similar additional language courses in different fields.

In the UCT MBChB degree, the programme is one that integrates the learning of the languages within the content of clinical courses across several years. This study advocates that other institutions continue to follow suit by incorporating the predominant additional languages in their respective provinces.

Interns in our study confirmed recommendations in the study by Mohamed, Roche et al., (2019) that additional contact periods could be offered instead of just one weekly contact session of one-and-a-half hours.

In pursuance of a more rounded skill set, the course convenors and designers explored the possibility of addressing gaps in the *Languages* curriculum. One such initiative is the compulsory SSM, which commenced in 2009. The SSM should be strengthened and expanded to include a larger cohort of future students.

Additional variables that need acknowledgment and attention in the existing course in relation to the speed of developing proficiency in a language, include the effect of fostering an attitude of willingness to learn a new language and creating a culture of self-directed learning by foregrounding the necessity and relevance of consultations in the patient’s mother tongue.

Some interns, who are beginners in the target languages continue to grapple with the retention of grammar and vocabulary that they had learned during their undergraduate years. A solution to this matter would be to tweak the existing curriculum to the extent that it offers a blend of verbal and embodied communication to establish the primary presenting complaint with certainty. Mastery of this can be tested during regular Objective, Structured Practical Examination type assessments (OSPE’s) which are conducted throughout the academic year.

Most of the interns who participated in this study did not have access to multimedia materials produced in Afrikaans, English and isiXhosa, which were developed in 2010 to supplement their classroom learning. These electronic materials are available on an Open Educational Resource, on OpenUCT (Clinical Skills Examination Procedures: Afrikaans and isiXhosa (https://vula.uct.ac.za/x/5lgphv), where current and past students have access to these practical learning aids that bolster discipline-specific communication skills. Tools like this could be combined with a future in-class focus on dyadic communication in both languages. Interns would not only understand patient responses with greater ease but would continue to deepen overall communicative abilities and cultural understanding as they interact with their patients.

## Conclusion

The findings of this study provide insight into the degree of perceived communication competency in the additional languages that is achieved in the authentic workplace context. The study has shown that the usefulness on each of the probed categories was not consistent across both languages. One of the contributing factors leading to this inconsistency was identified as the non-cognate relationship between Afrikaans and isiXhosa in respect of syntactic, morphological and semantic relations.

The results also confirm a misalignment between the languages that are most spoken by doctors and those spoken by patients, as determined in previous studies. Often patients are more likely to speak a language other than Afrikaans, English and isiXhosa during their encounter with a doctor.

Interns report understanding the cultural context related to isiXhosa better than for Afrikaans. They can greet patients fluently (equally in both languages), and understand their replies, more so for Afrikaans than for isiXhosa.

Relative to Afrikaans, a larger proportion of interns indicated an inability, or uncertainty, to establish the presenting complaint in isiXhosa.

The interns can ask relevant questions to obtain a history from a patient in Afrikaans and understand patient responses to their questions, but to a lesser extent in isiXhosa during clinical examinations. Interns could also give patients the necessary instructions and were mostly able to understand their replies better in Afrikaans, than in isiXhosa.

Of the interns who responded more than half (54%) were confident in their ability to counsel patients on their illness in Afrikaans, while less than half (17%) reported the same for isiXhosa.

Interns possess the terminology to discuss types of medication, how frequently it should be used and what its indications are. Twice as many interns were confident to explain in Afrikaans how to use their medication compared to isiXhosa.

The results in the category for health promotion suggest that this is one area in the BaDr communications course that requires attention. There is thus doubt whether an investment of one contact session per week is sufficient to ensure appropriate growth and development for students to reach levels of the additional language proficiency.

Overall, qualitative focus group findings indicated that the knowledge of even the basics of the target languages were beneficial to interns within their present clinical environments.

## Data Availability

The data sets used and/or analised during the current study are available from  10.25375/uct.24003798.v1.

## Abbreviations

UCT: University of Cape Town
MBChB: Bachelor of Medicine and Bachelor of Surgery degrees
BaDr: Becoming-a-Doctor
HPCSA: Health Professions Council of South Africa
WC: Western Cape
SSM: Special Study Module
CHC: Community Healthcare Centre
ACTFL: American Council on the Teaching of Foreign Languages
DLPT: Defence Language Proficiency Test
ILR: Interagency Language Roundtable scale
